# Notch Signaling Pathway Is Inhibited in the Development of Barrett's Esophagus: An In Vivo and In Vitro Study

**DOI:** 10.1155/2018/4149317

**Published:** 2018-03-26

**Authors:** Yun-Cang Wang, Zhi-Qiang Wang, Yong Yuan, Tao Ren, Peng-Zhi Ni, Long-Qi Chen

**Affiliations:** ^1^Department of Thoracic Surgery, West China Hospital, Sichuan University, Chengdu, China; ^2^Department of Thoracic Surgery, Hospital of Chengdu Office of People's Government of Tibetan Autonomous Region, Chengdu, Sichuan, China; ^3^Division of Gastroenterology, Hospital of Chengdu Office of People's Government of Tibetan Autonomous Region, Chengdu, Sichuan, China

## Abstract

**Objective:**

To explore the role of Notch signaling in the development of Barrett's esophagus.

**Methods:**

Patients with esophagectomy and gastric interposition were recruited as a human model of gastroesophageal reflux disease. The expressions of Notch signaling genes in normal esophagus from surgical specimen and columnar metaplasia in the esophageal remnant after esophagectomy were evaluated by real time quantitative Polymerase Chain Reaction (RT-qPCR) and immunohistochemistry (IHC). For in vitro experiments, Het-1A cells were treated with hydrochloric acid, deoxycholic acid, mixture of hydrochloric acid and deoxycholic acid, or Notch1-siRNA, and expressions of Notch1, Hes1, MUC2, and K13 were evaluated via RT-qPCR and western blot.

**Results:**

Samples were obtained from 36 patients with columnar metaplasia in the esophageal remnant. Both IHC and RT-qPCR indicated that Notch1 and Hes1 expressions were significantly higher in normal esophagus than that in metaplasia. Hydrochloric acid and deoxycholic acid suppressed Notch1, Hes1, and K13 expressions, in concert with increasing MUC2 expressions. Notch inhibition by Notch1-siRNA contributed to the downregulation of Notch1, Hes1, and K13 expressions, whereas MUC2 expression was enhanced.

**Conclusions:**

Both hydrochloric acid and deoxycholic acid could suppress Notch signaling pathway in esophageal epithelial cells, and inhibited Notch signaling has important functions in the development of Barrett's esophagus.

## 1. Introduction

Barrett's esophagus (BE), a condition wherein metaplastic columnar epithelium replaces normal stratified squamous epithelium, is a consequence of chronic esophageal mucosal injury caused by gastroesophageal reflux disease (GERD) [1]. GERD and Barrett's esophagus have clinical importance because they confer major risk factors for esophageal adenocarcinoma, one of the most deadly cancers worldwide [2]. Unfortunately, it remains unclear how GERD induces the BE and what molecular mechanism is involved. It has been proposed that some key developmental transcription factors might be involved in the development of reflux-related mucosal injury and Barrett's esophagus [3, 4].

Notch signaling pathway, a necessary intercellular signaling pathway for early development of multiple tissues and organs, is widely involved in regulating cell development, proliferation, differentiation, and apoptosis. It is generally regarded as an important signaling pathway for cell fate determination [5]. Some studies have shown that Notch signaling pathway regulates intestinal epithelial differentiation and decides the destiny and final outcomes of the intestinal epithelial cells. The Notch-knockout mice or the use of *γ*-secretase inhibitor will induce the metaplastic change of the goblet cells and inhibit the proliferation of intestinal epithelial cells, which might present as novel therapeutic target [6, 7]. A recent study compared the genome-wide expression in Barrett's esophagus and normal esophageal epithelium. The result showed that most of the genes related to the Notch signaling pathway were downregulated in Barrett's esophagus than in the normal esophageal epithelium [8]. Nevertheless, the role of Notch in the development of Barrett's esophagus is still controversial, which has not been systematically investigated both in vivo and in vitro experiment.

Esophagectomy with gastric interposition is usually indicated for patients with resectable esophageal cancer. Nevertheless, the normal antireflux mechanisms have to be damaged during operation. Patients with esophagectomy would inevitably suffer from significant reflux symptoms and reflux-related esophageal mucosal damage would eventually occur [9, 10]. Accordingly, esophagectomy and gastric interposition serve as an ideal human reflux model to study the molecular pathogenesis of reflux-induced esophageal mucosal damage. In the present study, we utilized this model to investigate the role of Notch signaling in the development of Barrett's esophagus. In vitro study was also conducted to explore the potential role of Notch pathway in Barrett's esophagus.

## 2. Materials and Methods

### 2.1. Study Population and Sample Preparation

This work was approved by the ethics committee of West China Hospital and informed consent was obtained from all patients. All experiments were performed in accordance with the relevant guidelines and regulations. From February 2011 to February 2016, patients with esophagectomy and gastric interposition for esophageal cancer were selected for upper gastrointestinal endoscopy and biopsies. We only included patients with newly diagnosis of columnar metaplasia in the residual esophagus after esophagectomy. Patients with preoperative history of gastroesophageal reflux disease, preoperative/postoperative adjuvant therapy for cancer, or evidence of tumor recurrence during the follow-up period were excluded from this study.

During operation, two pieces of normal esophageal mucosa from resected specimen were routinely obtained, one was flash frozen for Polymerase Chain Reaction (PCR), and the other was fixed into formalin for immunohistochemistry (IHC). For the postoperative endoscopy, four circumferential biopsies were taken by conventional forceps from suspected area of the esophageal remnant or at a distance of 2 cm away from the anastomotic site when there was no damage visualized. Two biopsies were fixed in formalin immediately for hematoxylin and eosin (HE) staining IHC, and the other two were flash frozen by liquid nitrogen for RT-qPCR.

### 2.2. Immunohistochemistry

The protein expression of Notch signaling pathway related genes (Notch1, Hes1) in samples was detected by immunohistochemical method. IHC staining was performed using formalin-fixed paraffin-embedded blocks as previously described [11]. The following primary antibodies were used: anti-Notch1, Santa Cruz, 1 : 100 dilution; anti-Hes1, Abcam, 1 : 200 dilution. Known positive controls using normal skin tissue were included for each run, and negative controls were done by omitting the primary antibodies. Two independent observers assessed immunoreactivity using a three-grade system, where 0 denoted negative staining; 1 denoted minimal and variable staining; 2 denoted obvious and intense staining. Sections with grade 2 were considered positive staining.

### 2.3. Real Time Quantitative Polymerase Chain Reaction (RT-qPCR)

RT-qPCR was performed to detect mRNA expression levels of target genes in samples as previously described [12]. Trizol reagent (Invitrogen) was used to extract total RNA and complementary DNA (cDNA) was prepared using the QuantiTech Reverse Transcription kit (Qiagen). The RT-PCR was performed on Rotor Gene 3000 by using QuantiTect SYBR Green PCR kit, according to the instructions. *β*-Actin was used as reference gene, and results were expressed as the relative expression ratio of target gene to reference gene. The sequences and amplicon size for the primers are listed in Table 1.

### 2.4. Effects of Hydrochloric Acid and Deoxycholic Acid on the Notch Signaling Pathway

Human esophageal squamous cell line Het-1A (normal human esophageal squamous epithelial cell line immortalized by viral SV40 transfection) was purchased from China Center for Type Culture Collection (Wuhan, China). The cells were grown under standard conditions and treated with medium containing different concentrations of hydrochloric acid (pH4, pH5, and pH6), deoxycholic acid (DCA, 300 umol/L, 500 umol/L, and 1000 umol/L) or mixture of both (hydrochloric acid pH5 + DCA 500 umol/L), and blank controls were set up. Then cells were harvested at different time of incubation (24 h, 48 h, 72, and 96 h). Cell incubation time, hydrochloric acid, deoxycholic acid, and concentrations were chosen with reference to studies described elsewhere [12, 13]. Het-1A cells (5,000 cells/well) were seeded into 96-well plates and stimulated with hydrochloric acid (pH4, pH5, and pH6), deoxycholic acid (DCA, 300 umol/L, 500 umol/L, 1000 umol/L), or mixture of both (hydrochloric acid pH5 + DCA 500 umol/L). MTT analysis was used to detect cell viability at 596 nm at 24, 48, 72, and 96 h after stimulation. Notch signal genes (Notch1 and downstream target Hes1), goblet cell-specific gene Mucin 2 (MUC2), and squamous keratin related gene (K13) were detected by RT-qPCR and western blot analysis as previously reported [14]; the antibodies used for western blotting are summarized in Table 2.

### 2.5. Small Interfering RNA (siRNA) Knockdown of Notch Signaling

The siRNA was used to silence Notch1 expression. The Notch1-siRNA was synthesized by Obio Technology (Shanghai, China). The sequences were as follows: 5′-GATCCTGGCGGGAAGTGTGAAGCGT-3′, 5′-AGACGCTTCACACTTCCCGCCATTA-3′. And the random sequences were used as negative control. Het-1A cells were transfected with Notch1-siRNA by using Lipofectamine 2000 according to the manufacturers' instructions. After incubation, cells were harvested and analyzed by RT-PCR and western blot as previously described [14].

### 2.6. Data Analysis

The measurement data with normal distribution were described as the mean ± standard deviation; otherwise they were expressed as median with interquartile range. The normal distribution data among multigroups were compared using single factor analysis of variance (ANOVA), and independent samples *t*-test was used to compare differences between groups. For the data with the nonnormal distribution, the Kruskal-Wallis test was used among multigroups and the Mann–Whitney *U* test was applied to compare differences between groups. Fisher's test was applied to compare categorical data between groups. The correlation between each gene was analyzed by Spearman correlation analysis. Two sides *p* < 0.05 were considered to be statistically significant, and data were processed using SPSS 19.0 (IBM, Inc., Chicago, Illinois, USA).

## 3. Results

### 3.1. Characteristics of Patients

From February 2011 to February 2016, 36 postesophagectomy patients with histologically confirmed columnar metaplasia in the esophageal remnant were included. All patients underwent esophagectomy with gastric interposition for esophageal squamous cell carcinoma. The mean follow-up period was 4.7 years. The main clinical characteristics of the included patients are shown in Table 3.

### 3.2. Immunohistochemical Analysis for Normal Esophageal Epithelium and Columnar Metaplasia

**Figure 1:**
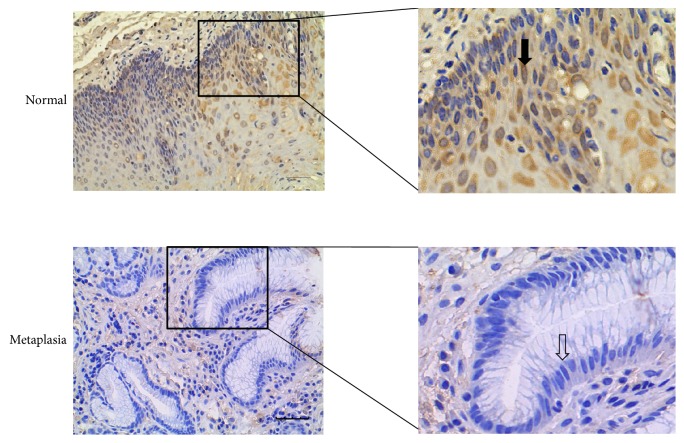
Representative Notch1 immunohistochemical staining in samples with different histological alterations. Notch1 protein was mainly expressed in cytoplasm of esophageal cells. Intense staining of Notch1 was mainly present in normal esophagus, and metaplastic columnar cells mainly exhibited negative staining (arrows). Scale bar in the figure is 40 *μ*m. Magnified figures were shown in the right side.

**Figure 2:**
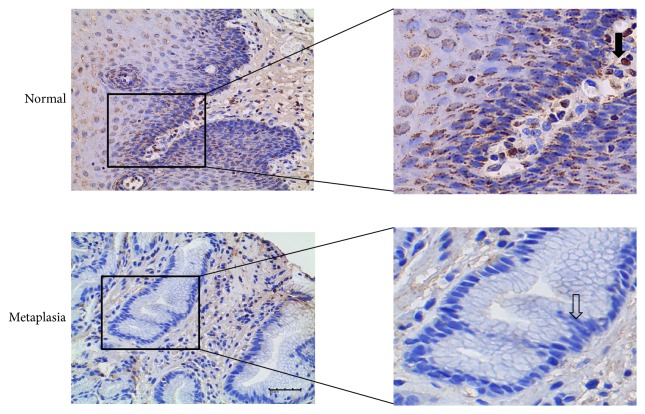
Representative Hes1 immunohistochemical staining in samples with different histological alterations. Intense Hes1 protein was mainly expressed in cytoplasm and nucleus of normal esophageal cells, and metaplastic columnar cells mainly exhibited negative staining (arrows). Scale bar in the figure is 40 *μ*m. Magnified figures were shown in the right side.

Normal esophageal mucosa from surgical specimen and columnar metaplastic samples in the esophageal remnant after esophagectomy were subject to IHC for Notch1 and Hes1. Histological evaluation of esophageal mucosa was done on sections with hematoxylin and eosin staining by independent pathologists. The results and representative pictures for immunohistochemical staining are shown in Tables 4 and 5, Figures 1 and 2. Notch1 protein was mainly expressed in cytoplasm of esophageal cells. The expression of Notch1 protein in normal esophageal epithelium was higher than that in metaplastic tissue (*p* < 0.001). As the Notch signaling downstream target gene, Hes1 expression was mainly located in cytoplasm and nucleus. It was significantly higher in normal esophagus than in metaplastic tissue (*p* < 0.001), which was in accordance with expression trend of Notch1. Furthermore, the protein expression of Notch1 was positively correlated with Hes1 protein expression (*p* < 0.001).

### 3.3. Real Time Quantitative Polymerase Chain Reaction (RT-qPCR) for Normal Esophagus, and Metaplastic Tissues

**Figure 3:**
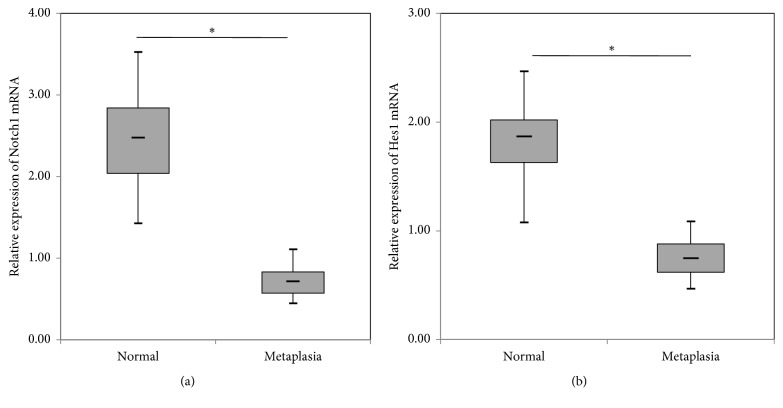
Relative expression of Notch1 (a) and Hes1 (b) mRNA in normal esophagus and metaplastic esophagus. Significant differences were detected in Notch1 mRNA and Hes1 mRNA expression values between normal squamous mucosa and metaplastic tissue. ^*∗*^*p* < 0.05, statistically significant difference.

To analyze Notch1 and Hes1 mRNA expression levels in samples with different histological alterations, RT-qPCR was performed (Figure 3). Notch1 mRNA expression level in normal esophageal epithelium was higher than that in columnar metaplastic tissues (*p* < 0.001). We also found a decreasing expression pattern of Hes1 mRNA from normal esophagus to columnar metaplasia (*p* = 0.017). Furthermore, Notch1 and Hes1 mRNA expressions were positively correlated (*p* = 0.028).

### 3.4. Effects of Hydrochloric Acid and Deoxycholic Acid on Notch1, Hes1, K13, and MUC2 Expressions

**Figure 4:**
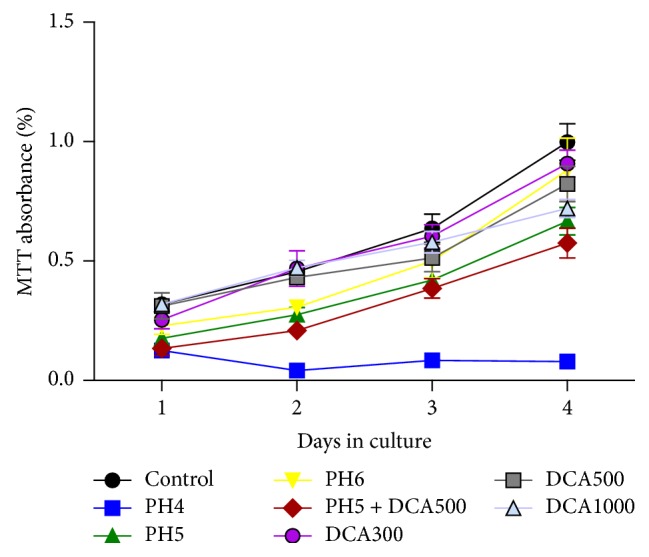
MTT assay was performed to detect cell viability at 24, 48, 72, and 96 h in Het-1A cells with different stimuli.

Hydrochloric acid and bile acid (deoxycholic acid) are known to play an important role in the development of reflux-related esophageal mucosal damage; we investigated their effects on Notch signaling (Notch1, Hes1), goblet cell-specific gene Mucin 2 (MUC2), and squamous keratin related gene (K13) expressions using Het-1A cells. Stimuli were added to the culture at different concentrations and indicated times. The cell proliferation was assessed by methyl thiazolyl tetrazolium (MTT) assay (Figure 4). The results showed cell proliferation was decreased with a more acidic pH. Het-1A cells could not survive when pH values are lower than 4. When the deoxycholic acid concentration was 300–400 umol/L, the cell proliferation rate decreased with the increasing deoxycholic acid concentration. From our study, it seemed that a prolonged incubation time of 3-4 days did not have negative effects on Het-1A cells. Concentration and pH of the stimuli were more important for cell viability and proliferation.

#### 3.4.1. Effects of Hydrochloric Acid on Notch Signaling

**Figure 5:**
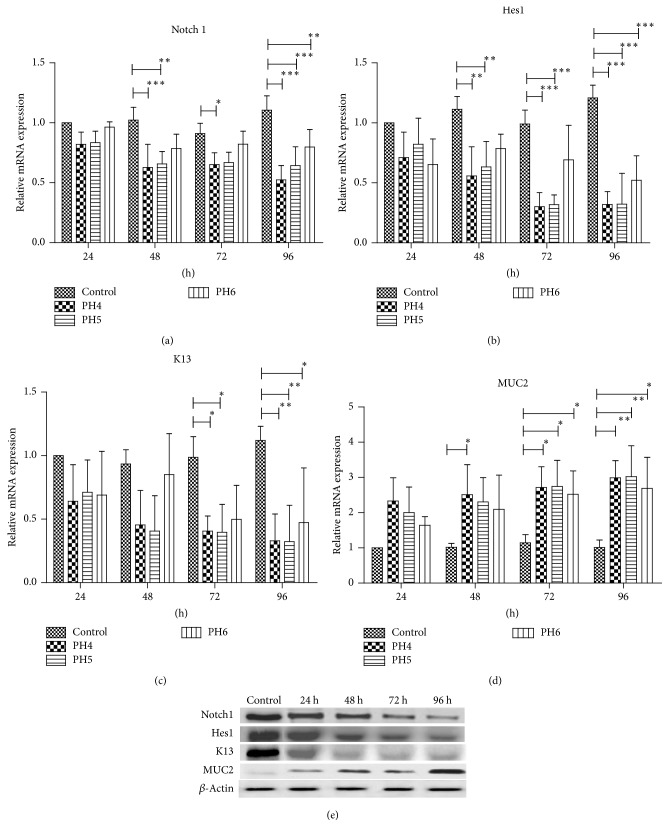
Effects of hydrochloric acid on Notch1, Hes1, K13, and MUC2 expressions. Cells were treated with different concentrations of hydrochloric acid (pH4, pH5, and pH6) for 24 h, 48 h, 72 h, and 96 h, respectively. (a–d) Notch1, Hes1, K13, and MUC2 mRNA expression levels in Het-1A cells exposing to different concentrations of hydrochloric acid for different time periods. ^*∗*^*p* < 0.05, statistically significant difference. ^*∗∗*^*p* < 0.01, statistically significant difference. ^*∗∗∗*^*p* < 0.001, statistically significant difference. (e) Representative western blot results of Notch1, Hes1, K13, and MUC2 protein expressions in Het-1A cells treated with hydrochloric acid (pH5) for different time periods.

Cells were stimulated with different concentrations of hydrochloric acid (pH4, pH5, and pH6); we found that Notch1 mRNA expression was suppressed in a concentration-dependent manner. And K13 mRNA expression was inhibited in a time-dependent manner, whereas Hes1 mRNA expression was decreased in concentration- and time-dependent manners. On the other hand, the expressions of MUC2 mRNA in Het-1A cells were increased in concentration- and time-dependent manners. We also investigated the effects of hydrochloric acid on Notch1, Hes1, MUC2, and K13 protein expressions, showing that expression patterns were consistent with their mRNAs. Representative pictures were shown in Figure 5.

#### 3.4.2. Effects of Deoxycholic Acid on Notch Signaling

**Figure 6:**
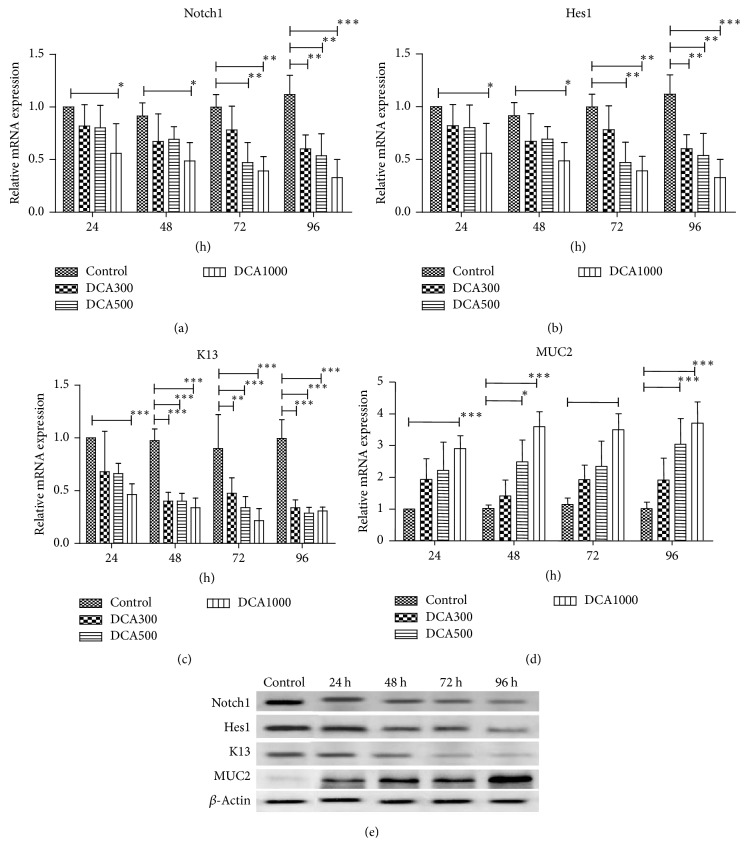
Effects of deoxycholic acid on Notch1, Hes1, K13, and MUC2 expressions. Cells were treated with different concentrations of deoxycholic acid (300 umol/L, 500 umol/L and 1000 umol/L) for 24 h, 48 h, 72 h, and 96 h, respectively. (a–d) Notch1, Hes1, K13, and MUC2 mRNA expression levels in Het-1A cells exposing to different concentrations of deoxycholic acid for different time periods. ^*∗*^*p* < 0.05, statistically significant difference. ^*∗∗*^*p* < 0.01, statistically significant difference. ^*∗∗∗*^*p* < 0.001, statistically significant difference. (e) Representative western blot results of Notch1, Hes1, K13, and MUC2 protein expressions in Het-1A cells treated with deoxycholic acid (500 umol/L) for different time periods.

When Het-1A cells were cultured with deoxycholic acid (300 umol/L, 500 umol/L, and 1000 umol/L), both PCR and WB indicated that Notch1, Hes1, and K13 expressions were suppressed in concentration- and time-dependent manners. However, MUC2 expressions were augmented in concentration- and time-dependent manners. Representative pictures were shown in Figure 6.

#### 3.4.3. Effects of Mixture of Hydrochloric Acid and Deoxycholic Acid on Notch Signaling

**Figure 7:**
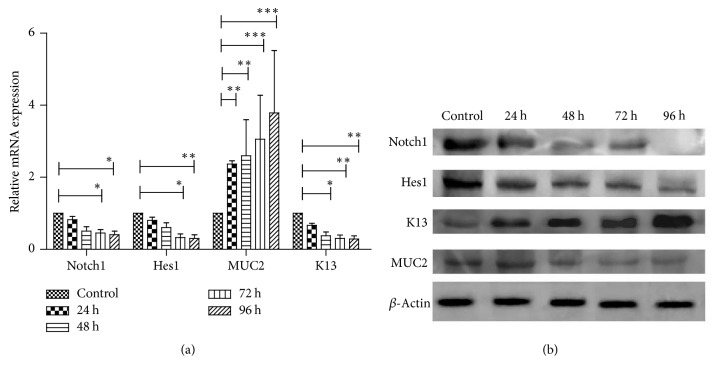
Effects of mixture of hydrochloric acid and deoxycholic acid on Notch1, Hes1, K13, and MUC2 expressions. Cells were treated with mixture of hydrochloric acid (pH = 5) and deoxycholic acid (500 umol/L) for 24 h, 48 h, 72 h, and 96 h, respectively. (a) Notch1, Hes1, K13, and MUC2 mRNA expression levels in Het-1A cells exposing to mixture of hydrochloric acid and deoxycholic acid for different time periods. ^*∗*^*p* < 0.05, statistically significant difference. ^*∗∗*^*p* < 0.01, statistically significant difference. ^*∗∗∗*^*p* < 0.001, statistically significant difference. (b) Representative western blot results of Notch1, Hes1, K13, and MUC2 protein expressions in Het-1A cells treated with mixture of hydrochloric acid (pH = 5) and deoxycholic acid (500 umol/L) for different time periods.

Both hydrochloric acid and deoxycholic acid may play important roles in development of reflux-related esophageal mucosa injury. We also evaluated effects of mixture of hydrochloric acid and deoxycholic acid on Notch signaling. According to our previous results, the mixture of hydrochloric acid (pH5) and deoxycholic acid (500 umol/L) was used for cell treatment. Our findings suggested that mixture of hydrochloric acid and deoxycholic acid could suppress Notch1, Hes1, and K13 expressions in a time-dependent manner, in concert with increasing MUC2 expressions. Representative pictures were shown in Figure 7.

### 3.5. Effects of Notch Signaling Inhibition

**Figure 8:**
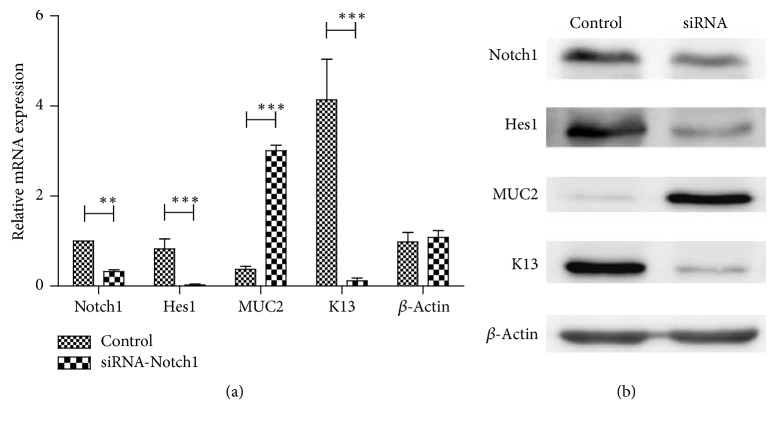
Effects of Notch signaling inhibition. Expressions of Notch1, Hes1, and K13 were significantly downregulated in cells transfected with Notch1-siRNA, in concert with significant increase of MUC2 expression. (a) Notch1, Hes1, K13, and MUC2 mRNA expression levels by RT-qPCR in Het-1A cells transfected with Notch1-siRNA. ^*∗∗*^*p* < 0.01, statistically significant difference. ^*∗∗∗*^*p* < 0.001, statistically significant difference. (b) Notch1, Hes1, K13, and MUC2 protein expressions by western blot analysis in Het-1A cells after Notch signaling inhibition by Notch1-siRNA.

In order to further investigate the effect of Notch knockdown on the expressions of goblet cell-specific gene MUC2 and squamous keratin related gene K13, Het-1A cells were used as in vitro models via Notch1-siRNA method. In our experiment, the Notch1 mRNA expression level showed a remarkable decrease in the Notch1-siRNA group as compared to that in control group. Western blot analysis also verified that Notch1 protein expression was significantly downregulated after Notch1-siRNA transinfection. In addition, the expressions of Notch1 downstream target gene (Hes1) and squamous keratin related gene (K13) were remarkably inhibited in Notch1-siRNA group as evaluated by RT-PCR and western blot. On the contrary, inhibition of Notch signaling by Notch1-siRNA contributed to a significant increase of MUC2 expression. Representative pictures were shown in Figure 8.

## 4. Discussion

Studies on BE cover a variety of topics, among which gastroesophageal reflux is one of the most researched and understood. Many believe that BE is the results of adaptive metaplasia in an acid environment. The origin of newly generated columnar cells is still much disputed. The mainstream opinion holds that columnar cells originate from differentiation of basal layer of the esophagus or differentiation of esophageal submucosal glandular cells. Others believe that the columnar cells originate from the upward migration of the cells at the gastroesophageal junction to the esophageal mucosa. Regardless of the cellular origin for BE, it is generally believed that the refluxed contents alter the microenvironment of esophageal epithelial cells, which further leads to BE [15, 16]. However, the key molecular pathogenesis of this process is largely unknown.

It has been well documented that Notch signaling plays an important role in cell fate determination [5]. Whether Notch signaling pathway is involved in the development of BE is still controversial, which has not yet been systematically investigated in vivo and in vitro experiments. In our study, we firstly included patients with esophagectomy and gastric interposition as an ideal human model of gastroesophageal reflux. Normal esophagus from surgical specimen represented normal esophageal mucosa without reflux-related injury and postesophagectomy metaplasia in residual esophagus developed following long-term exposure to reflux. Expressions of Notch1 and Hes1 were evaluated for the biopsy specimens, and the expression levels of Notch1 and Hes1 had no correlation with the age, gender of the patients, and location and staging of tumor. Expression of Notch signaling was compared between normal esophageal mucosa and reflux-related columnar metaplasia. Both immunohistochemistry and PCR analyses indicated that Notch1 expression levels were decreased from normal esophagus to columnar metaplasia. As the Notch signaling pathway downstream target gene, Hes1 expression exhibited the same trend. For the first time, evidence from this in vivo human reflux model suggested that Notch signaling might be suppressed in the development of BE. Notably, Notch signaling has been investigated in rat and mouse models. They demonstrated that Notch inhibition could induce goblet cell differentiation and reduce cell proliferation, while cellular proliferation and progression of BE could be promoted by Notch activation, suggesting that Notch signaling might play binary roles in regulating Barrett metaplasia and its progression [4]. In this human reflux model, we also found Notch inhibition in the development of BE metaplasia. However, it is still unclear if Notch plays a role in the progression of BE in this in vivo model. It would be very interesting to continue to follow up our patients for further exploring the role of Notch signaling on cellular proliferation and BE progression.

BE is considered to be caused by chronic gastroesophageal reflux. We further investigated effects of reflux contents on the expressions of Notching signaling via in vitro experiments. It has been confirmed that both gastric acid and bile acid are composed of the gastric contents refluxing up to the esophagus, though in varying proportions. The gastric fluid is usually mixed with bile, and both play a synergistic role in esophageal mucosal damage [17–19]. In the current study, esophageal cells (Het-1A) were stimulated with hydrochloric acid, deoxycholic acid, or mixture of the two. We found that both hydrochloric acid and deoxycholic acid suppressed Notch signaling (Notch1 and Hes1), and deoxycholic acid exhibited a stronger effect as compared with hydrochloric acid. Previously, Tamagawa et al. [19] evaluated expression and function of Notch signaling pathway in the development of BE. Firstly, they compared Notch expressions between human normal esophagus and BE, and then some esophageal cell lines were stimulated with bile acid or gamma-secretase inhibitor. They found Hes1 expression was significantly lower in BE than in normal esophageal specimens, with no significant difference between BE and normal esophagus for Notch1 expression. This result was further validated by the in vitro experiments using deoxycholic acid incubation (DCA 200 uM, 6–12 hours). However, both in vivo and in vitro experiments in our study revealed that expressions of Notch1 and Hes1 were decreased in the development of BE. One possible explanation for this discrepancy might be that Notch1 antibodies used in the experiments were different. Tamagawa et al. used Notch1 C-terminus antibody or cleaved Notch1 antibody which detects Notch1 intracellular domain and does not recognize extracellular domain. Besides, different cell culture and stimulation conditions might result in different gene expressions.

BE is featured by replacement of squamous cells with columnar cells, typically with the presence of goblet cells. Squamous cells contain squamous keratin and K13 is a common protein for detecting squamous keratin. Mucin 2 (MUC2) is considered specific to the goblet cells, and the secretory function of the cells can be characterized by MUC2 [7, 20–22]. In this experiment, MUC2 and K13 were used as gene markers for determining different functional inclinations of cells. Our findings indicated that both hydrochloric acid and deoxycholic acid would inhibit Notch signaling, in concert with increased expression of MUC2 and downregulated K13 expression, implying that Notch signaling inhibition by hydrochloric acid and bile acid could promote transdifferentiation of esophageal epithelial cells toward columnar-like cells.

To further evaluate whether K13 downregulation and MUC2 upregulation were induced by Notch signaling inhibition, we employed treatment of Notch1-siRNA in Het-1A cells. Both PCR and WB confirmed effective inhibition of Notch1 expression by specific Notch1-siRNA. The Notch downstream target gene Hes1 was also decreased, in concert with inhibition of K13 expression and elevation of MUC2 expression. In addition, inhibition of Notch signaling was correlated well with K13 downregulation and MUC2 upregulation. Consistent with our study, Tamagawa et al. used gamma-secretase inhibitor to inhibit Notch signaling in human esophageal cells and revealed that Hes1 expression was suppressed, while MUC2 expression was augmented following stimulation with gamma-secretase inhibitor [19]. These findings indicated that Notch signaling inhibition has the potential role to promote transdifferentiation of esophageal epithelial cells toward columnar-like cells as demonstrated by increased expression of glandular Mucin (MUC2) and decreased expression of squamous keratin (K13). One limitation for our in vitro study which needs to be mentioned is that only one cell line was investigated, two or more cell lines need to be further investigated to verify the findings. In Krishnadath's study, they compared gene expression profile for the BE and squamous esophagus by serial analysis of gene expression (SAGE), they found 72 tags were more than 10-fold up-regulated, and 26 tags were more than 10-fold downregulated. But they did not report a decreased expression of Notch signaling [23]. However, another study by Vega et al. also performed Affymetrix gene expression microarray on BE tissues and showed decreased Notch signaling for BE samples. Furthermore, via 3D organotypic culture technique, they observed elongated cells in the basal layer of epithelium after inhibition of Notch signaling. And they concluded that esophageal epithelial transdifferentiation might promote the evolution of BE [7].

## 5. Conclusion

In conclusion, findings from this in vivo and in vitro study suggest that both hydrochloric acid and deoxycholic acid could suppress Notch signaling pathway in esophageal epithelial cells and inhibited Notch signaling has important functions in the development of Barrett's esophagus.

## Figures and Tables

**Table 1:** The sequences for the primers used for PCR.

Gene	Forward primer (5′→3′)	Reverse primer (5′→3′)	Tm	Amplicon size
*β*-Actin	GAAGATCAAGATCATTGCTCCT	TACTCCTGCTTGCTGATCCA	58	111 bp
Notch1	GCCACCACTGCGAGACCAACATCAA	AGGCAGAAGCAGAGGTAGGCGTTGT	68	100 bp
Hes1	CGTGCGAGGGCGTTAATACCGAGGT	GAGGTGCCGCTGTTGCTGGTGTAGA	69	385 bp
MUC2	AACACCCTGCTCGTCATC	CAAATGCTGGCATCAAAGTTGG	65	117 bp
K13	CGGGATGCTGAGGAATGGTT	CTGACGCTTCTTGGCGTCCT	60	110 bp

**Table 2:** Antibodies used for western blotting.

Antibody	Company	Dilution
Notch1	Santa Cruz (sc-23299)	1 : 1000
Hes1	Abcam (ab49170)	1 : 2000
MUC2	Abcam (ab11197)	1 : 2000
K13	Abcam (ab92551)	1 : 1000
*β*-Actin	Abcam (ab8226)	1 : 5000

**Table 3:** Characteristics of patients.

Clinical characteristics	
Patients	*n* = 36
Age (years)	64 (51–68)
Gender	
Male	27 (75%)
Female	9 (25%)
Location of tumor	
Upper	3 (8%)
Middle	25 (69%)
Lower	8 (22%)
Pathological staging	
TisN0M0	5 (14%)
T1N0M0	9 (25%)
T2N0M0	22 (61%)
Adjuvant therapy	
None	36 (100%)
Yes	0 (0%)

**Table 4:** Immunohistochemical results of Notch1.

Group	*n*	IHC		*p*
		Positive	Negative	
Normal	36	31	5	<0.001
Barrett	36	7	29	

**Table 5:** Immunohistochemical results of Hes1.

Group	*n*	IHC		*p*
		Positive	Negative	
Normal	36	28	8	<0.001
Barrett	36	10	26	
